# Rapid and Robust Identification of Sepsis Using SeptiCyte RAPID in a Heterogeneous Patient Population

**DOI:** 10.3390/jcm13206044

**Published:** 2024-10-10

**Authors:** Robert Balk, Annette M. Esper, Greg S. Martin, Russell R. Miller, Bert K. Lopansri, John P. Burke, Mitchell Levy, Richard E. Rothman, Franco R. D’Alessio, Venkataramana K. Sidhaye, Neil R. Aggarwal, Jared A. Greenberg, Mark Yoder, Gourang Patel, Emily Gilbert, Jorge P. Parada, Majid Afshar, Jordan A. Kempker, Tom van der Poll, Marcus J. Schultz, Brendon P. Scicluna, Peter M. C. Klein Klouwenberg, Janice Liebler, Emily Blodget, Santhi Kumar, Xue W. Mei, Krupa Navalkar, Thomas D. Yager, Dayle Sampson, James T. Kirk, Silvia Cermelli, Roy F. Davis, Richard B. Brandon

**Affiliations:** 1 Rush Medical College and Rush University Medical Center, Chicago, IL 60612, USA; jared_greenberg@rush.edu (J.A.G.); mark_a_yoder@rush.edu (M.Y.); gourang_p_patel@rush.edu (G.P.); 2 Grady Memorial Hospital and Emory University School of Medicine, Atlanta, GA 30322, USA; aesper@emory.edu (A.M.E.); greg.martin@emory.edu (G.S.M.); jkempke@emory.edu (J.A.K.); 3 FirstHealth of the Carolinas, Pinehurst, NC 28374, USA; rmiller@firsthealth.org; 4 Intermountain Medical Center, Murray, UT 84107, USA; bert.lopansri@imail.org (B.K.L.); john.burke@imail.org (J.P.B.); 5 School of Medicine, University of Utah, Salt Lake City, UT 84132, USA; 6 Warren Alpert Medical School, Brown University, Providence, RI 02912, USA; mitchell_levy@brown.edu; 7 School of Medicine, Johns Hopkins University, Baltimore, MD 21205, USA; rrothma1@jhmi.edu (R.E.R.); vsidhay1@jhmi.edu (V.K.S.); 8 Pulmonary and Critical Care & Sleep Medicine, Department of Medicine, University of Miami, Miami, FL 33136, USA; fdalessio@miami.edu; 9 Anschutz Medical Campus, University of Colorado, Denver, CO 80045, USA; neil.aggarwal@cuanschuj.edu; 10 Loyola University Medical Center, Maywood, IL 60153, USA; emgilbert@lumc.edu (E.G.); jparada@lumc.edu (J.P.P.); 11 School of Medicine and Public Health, University of Wisconsin, Madison, WI 53705, USA; mafshar@medicine.wisc.edu; 12 Amsterdam UMC, University of Amsterdam, 1105 AZ Amsterdam, The Netherlands; t.vanderpoll@amsterdamumc.nl; 13 Division of Cardiothoracic and Vascular Anesthesia and Intensive Care Medicine, Department of Anesthesia, General Intensive Care, and Pain Management, Medical University of Vienna, 1090 Vienna, Austria; marcus.j.schultz@gmail.com; 14 Nuffield Department of Medicine, University of Oxford, Oxford OX1 2JD, UK; 15 Centre for Molecular Medicine and Biobanking, University of Malta, Msida MSD 2080, Malta; brendon.scicluna@um.edu.mt; 16 Department of Applied Biomedical Science, Faculty of Health Sciences, Mater Dei Hospital, University of Malta, Msida MSD 2080, Malta; 17 Fundashon Mariadal, Kralendijk, Bonaire, Netherlands Antilles; pkleinklouwenberg@fundashonmariadal.org; 18 Keck Hospital of University of Southern California (USC), Los Angeles, CA 90033, USA; liebler@usc.edu (J.L.); eblodge1@hs.uci.edu (E.B.); santhi.kumar@med.usc.edu (S.K.); 19 Los Angeles General Medical Center, Los Angeles, CA 90033, USA; 20 Princeton Pharmatech, Princeton, NJ 08540, USA; winnie.mei@princetonpharmatech.com; 21 Immunexpress Inc., Seattle, WA 98109, USA; krupa.n@immunexpress.com (K.N.); dayle.s@immunexpress.com (D.S.); james.k@immunexpress.com (J.T.K.); silvia.c@immunexpress.com (S.C.); roy.d@immunexpress.com (R.F.D.)

**Keywords:** sepsis, SIRS, sepsis likelihood, SeptiCyte, host immune response, stratification, phenotype

## Abstract

**Background/Objective:** SeptiCyte RAPID is a transcriptional host response assay that discriminates between sepsis and non-infectious systemic inflammation (SIRS) with a one-hour turnaround time. The overall performance of this test in a cohort of 419 patients has recently been described [Balk et al., J Clin Med 2024, 13, 1194]. In this study, we present the results from a detailed stratification analysis in which SeptiCyte RAPID performance was evaluated in the same cohort across patient groups and subgroups encompassing different demographics, comorbidities and disease, sources and types of pathogens, interventional treatments, and clinically defined phenotypes. The aims were to identify variables that might affect the ability of SeptiCyte RAPID to discriminate between sepsis and SIRS and to determine if any patient subgroups appeared to present a diagnostic challenge for the test. **Methods:** (1) Subgroup analysis, with subgroups defined by individual demographic or clinical variables, using conventional statistical comparison tests. (2) Principal component analysis and k-means clustering analysis to investigate phenotypic subgroups defined by unique combinations of demographic and clinical variables. **Results:** No significant differences in SeptiCyte RAPID performance were observed between most groups and subgroups. One notable exception involved an enhanced SeptiCyte RAPID performance for a phenotypic subgroup defined by a combination of clinical variables suggesting a septic shock response. **Conclusions:** We conclude that for this patient cohort, SeptiCyte RAPID performance was largely unaffected by key variables associated with heterogeneity in patients suspected of sepsis.

## 1. Introduction

Accurate and rapid identification of sepsis is often clinically challenging, in part due to non-specific presenting clinical signs [1,2], patient heterogeneity [3,4,5], and a lack of timely information [6]. Clinical signs of sepsis are often vague and can include dyspnea, weakness, altered mental status, pain, and cough, which are signs commonly associated with other disease conditions, including heart failure, stroke, and respiratory failure [1,2].

To enable better identification and personalized treatment for sepsis, efforts have been made to identify subclasses of patients based on “phenotypes” [3,7,8] or “endotypes” [9,10,11]. For example, Seymour et al. [3] described four phenotypes differentiated by clinical parameters such as vasopressor requirement, age, chronic illness, renal dysfunction, inflammation, pulmonary dysfunction, shock, and liver dysfunction. As another example, Sinha et al. [8] showed that the “hyperinflammatory” and “hypoinflammatory” phenotypes previously identified in ARDS patients could also be applied to septic shock patients. Categorizing sepsis patients into clinically relevant phenotypes could potentially lead to personalized treatments (precision medicine) and better outcomes [12]. However, the successful application of precision medicine in sepsis assumes that this condition can be identified early and accurately in the first place. Traditional sepsis diagnostics that rely on the isolation and/or identification of causative pathogens, such as blood culture, have been shown to lack sensitivity and timeliness and, in general, have not taken patient heterogeneity into account [13]. More recent efforts to improve sepsis diagnosis have included the identification of host immune response biomarkers [14].

SeptiCyte RAPID is a host immune response test that measures mRNA expression levels of two genes, PLAC8 and PLA2G7, using a small peripheral blood sample [15,16]. The results are reported as a “SeptiScore” on a scale of 0–15 and in four “Bands”, with an increasing likelihood of sepsis associated with higher SeptiScores and bands. The discovery of these biomarkers was achieved via machine learning on a heterogeneous patient dataset [17]. Because the signature discovery process was performed on a heterogeneous set of patients, we hypothesized that the SeptiCyte RAPID signature would continue to perform robustly in independent heterogeneous validation datasets.

In the present study, as a test of the above hypothesis, we investigated the performance of SeptiCyte RAPID in a heterogeneous, critically ill adult patient cohort stratified by demographics, comorbidities and diseases, sources and types of infecting pathogen, therapeutic interventions, and clinical phenotypes. We sought to determine if the performance of SeptiCyte RAPID for differentiating sepsis from infection-negative systemic inflammatory response syndrome (SIRS) was robust and generalizable across different patient subgroups. The subgroups examined included patients with conditions that (1) present with overlapping clinical signs of sepsis, (2) may predispose to sepsis, and (3) could affect the performance of a host immune response assay.

## 2. Methods

### 2.1. Dataset and Comparator

The patient cohort and dataset for this analysis (*N* = 419 patients) were compiled from the MARS, VENUS, and NEPTUNE studies [15,16]. We have described the cohort in detail in our two previous publications and supplements therein [15,16]. Stratifications were performed according to sex, age, race/ethnicity, comorbidities and diseases, therapeutic interventions, and clinically defined phenotypes. The performance of SeptiCyte RAPID for discrimination of sepsis vs. SIRS was evaluated using Retrospective Physician Diagnosis (RPD) as the comparator. The RPD process is a clinical evaluation by a panel of three expert clinicians not involved in the care of the patients [15,16]. In this study, only “forced” RPD was used. That is, if a patient was initially called “indeterminate” by the RPD panelists, the panelists were then forced to make a consensus or unanimous call of sepsis or SIRS.

### 2.2. Statistical Analyses

#### 2.2.1. Conventional Statistical Tests

Conventional statistical tests were mainly conducted with the R ‘stats’ package or Medcalc (medcalc.org). Additional details and cross-checks were as follows. *p*-values for two-group comparisons were calculated with the Wilcoxon–Mann–Whitney test as implemented in the R ‘stats’ package [18] unless otherwise noted. Some *p*-value calculations for small *N* strata were conducted with Student’s *t*-test as implemented in Microsoft Excel (version 16.16.27) and also with the Mann–Whitney U test as implemented in Medcalc (version 20.104; medcalc.org) and cross-checked with the web applet at www.socscistatistics.com/tests/mannwhitney/default2.aspx (accessed on 10 August 2024). Proportions tests were conducted with the R ‘stats’ package and cross-checked with Medcalc. One-way ANOVA was conducted with the R ‘stats’ package. Cohen’s kappa was calculated with the web applet at http://vassarstats.net/kappa.html (accessed on 10 August 2024).

#### 2.2.2. Receiver Operating Characteristic (ROC) Curve Analysis

Receiver Operating Characteristic (ROC) curve analysis with calculation of area under curve (AUC) values was performed using the pROC package in R [19] and cross-checked with JROCFIT [http://www.rad.jhmi.edu/jeng/javarad/roc/JROCFITi.html, accessed on 15 August 2024] or Medcalc (medcalc.org). Confidence intervals for AUC were calculated by the Binomial Exact method, the method of Hanley and McNeil [20], the method of DeLong et al. [21] as implemented in pROC [19], or by bootstrapping as implemented with the web applet of Skalsk’a and Freylich [22] at http://www.freccom.cz/stomo/input.php (accessed on 19 August 2024). The Hanley and McNeil CI values were cross-checked with the web applet at https://riskcalc.org/ci/ (accessed on 19 August 2024). Note that the ROC analysis of small sample sets (AUC and 95% CI) relies on the equivalence relation AUC = U/(n_1_ × n_2_) where U = the Mann–Whitney U statistic and n_1_, n_2_ are the sizes of the SIRS and sepsis groups (see refs. [23,24] and Supplementary Material, Section S6). This approach for AUC calculation, utilized as a default by the DeLong’s implementation in the pROC package, is non-parametric and, hence, works better than parametric estimation for small sample sizes. AUC comparisons were performed with either DeLong’s test or the bootstrap method, as implemented in the pROC package in R [19]. The DeLong’s test calculations employed the roc.test function in the pROC package, which evaluates the difference between AUCs using Welch’s *t*-test. This approach assumes the two AUCs being compared are derived from patient groups having approximately normal distributions but allows for unequal sample sizes and unequal variances between the two patient groups. The assumption of normality for small *N* groups (<70 patients/group) was confirmed with the Kolmogorov–Smirnov and Shapiro–Wilk tests [25].

### 2.3. Bioinformatics Analyses

#### 2.3.1. Principal Components Analysis (PCA) + Hierarchical Clustering (HC)

Principal components analysis (PCA) + hierarchical clustering (HC) was performed using the FactoMineR package in R [26]. In the first stage of the analysis (PCA), 16 quantitative clinical variables were selected to define the dimensions (vectors) by which patients were separated in n-dimensional space. These variables, all measured within the first 24 h of ICU admission, were temperature (min and max), heart rate (HR; min and max), respiratory rate (RR; min and max), mean arterial pressure (MAP; min and max), glucose (min and max), white blood cells (WBC; min and max), platelets (min), procalcitonin (PCT), lactate, and age. Each variable had ≤33% missing data, and missing values were replaced with global mean values before conducting the analysis. (This is the default imputation method used by FactoMineR.) Additional variables were considered initially but excluded since they had >33% missing values each. SeptiScore and RPD categories were not used in the analysis.

The following qualitative or ordinal variables (not used in constructing the PCA dimensions) were also mapped onto the PCA plot: sex, vasopressor use, mechanical ventilation, pathogen type (bacterial, viral, fungal) and source of infection (blood, urine, sputum, or other), SOFA score, SOFA component scores, and qSOFA and component scores. Missing values for the ordinal variables SOFA, SOFA components, and qSOFA were replaced with global mean values, as for the quantitative variables. In the second stage of the analysis, HC was performed upon the PCA according to the method described in refs. [27,28,29] and implemented in FactoMineR. This allowed the PCA-based separation of patients to be further stratified into subgroups based on combinations of all the variables (quantitative, qualitative, and ordinal) defined above.

To verify the robustness of this analysis, we repeated the PCA + HC using group mean imputation instead of global mean imputation. In this case, groups were defined as either sepsis or SIRS by forced RPD. There was a very high degree of agreement between the two imputation methods in the proportions of samples in the clusters identified by PCA + HC. This is indicated in the Table 1 below.

#### 2.3.2. k-Means Clustering

k-means clustering [30], as implemented in the R ‘stats’ package, was applied to either the sepsis group (*N* = 176; results in the main text) or to the entire cohort (Sepsis+SIRS) (*N* = 419; results in Supplementary Material). The silhouette method [31] was used to determine the optimal number of clusters or subgroups. Many of the same variables were used for both the PCA analysis and the k-means analysis, including temperature (min and max within 24 h), heart rate (HR; min and max), respiratory rate (RR; min and max), mean arterial pressure (MAP; min and max), glucose (min and max), white blood cell count (WBC, min and max), platelets (min), procalcitonin (PCT), lactate, SOFA score, individual SOFA component scores, qSOFA score, age, sex, vasopressor use (Y/N), mechanical ventilation (Y/N), culture/PCR result (+/−) for bacterial, viral or fungal pathogen, and source of positive culture/PCR result (urine, blood, sputum, other). To impute missing data for each variable, the overall mean value for that variable was used. Prior to clustering, the continuous variables were transformed to have a mean of zero and a standard deviation of one. SeptiScore and RPD categories were not used as input variables.

## 3. Results

### 3.1. Demographics

Patient stratification was performed according to sex, age, and race/ethnicity. Patient ages ranged from 18 to 90 years and were binarized into subgroups <60 years or ≥60 years of age. The performance of SeptiCyte RAPID for discriminating sepsis vs. SIRS never fell below AUC 0.80 for any subgroup. For each demographic comparison, the *p*-value for discriminating sepsis vs. SIRS was significant at *p* < 0.05. When considered together in a multiple comparison context, the *p*-values all remained significant (*p* < 0.05/8) after applying a Bonferroni correction. No significant AUC differences were observed between subgroups except for a marginal difference for White vs. Hispanic (*p* = 0.03) (Table 2).

Upon further analysis, a significant White vs. Black difference in SeptiScore performance (*p* < 0.003) was observed for the discrimination of septic shock vs. SIRS. For this stratification, the White demographic subgroup gave AUC = 0.83, while the Black subgroup gave AUC = 0.96. Potential reasons for this observed difference are provided in the “Demographics” subsection of the Discussion. Further analysis is also provided in Supplementary Material, Section S1.

### 3.2. Comorbidities and Disease

**Figure 1:**
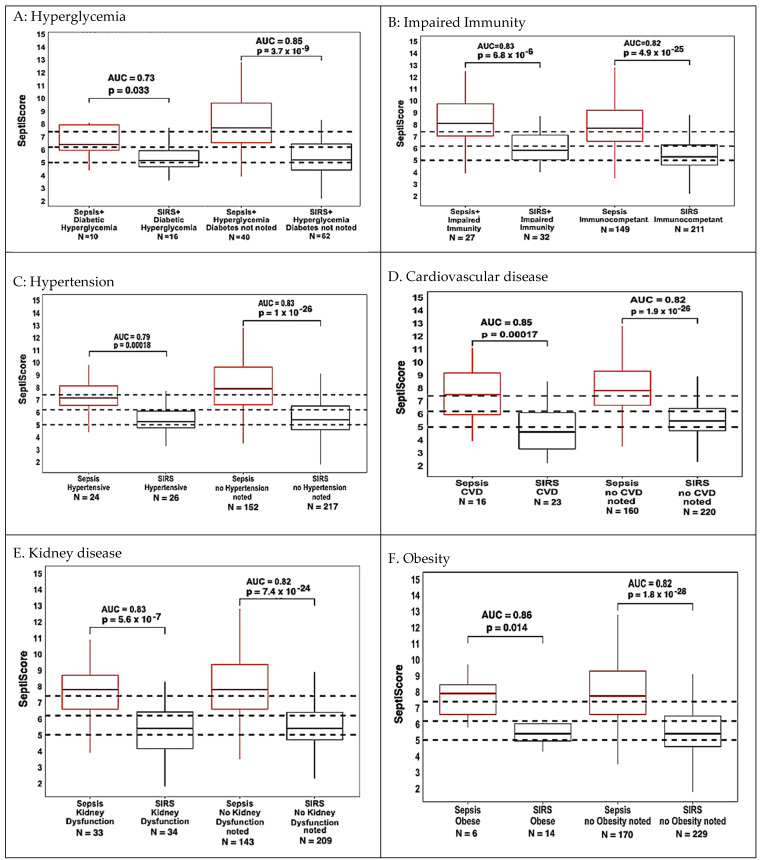
Box and whisker plots of SeptiCyte RAPID performance in patients with (**A**) hyperglycemia, (**B**) impaired immunity, (**C**) hypertension, (**D**) cardiovascular disease, (**E**) kidney disease, and (**F**) obesity. The dotted horizontal lines indicate the SeptiScore band boundaries as defined by Balk et al. [16].

Patients were stratified by the presence or absence of different comorbidities or diseases, specifically hyperglycemia, impaired immunity, hypertension, cardiovascular disease, kidney disease, and obesity. These comorbidities and diseases were chosen for analysis based on available patient numbers, presumed influence on sepsis predisposition, and potential for presenting clinical signs that overlap with those of SIRS or sepsis. The ‘impaired immunity’ category included patients with organic immune deficiencies, cancer patients on immunosuppressant drugs, and patients who received immunomodulators such as glucocorticoids; some patients fell into more than one sub-category. Patients for whom a particular comorbidity or disease was not mentioned in the physician notes were assumed to not have the condition. Point-estimate AUCs ranged from 0.81 to 0.86, with the only exceptions being AUC 0.79 for patients categorized as hypertensive and AUC 0.75 for patients with diabetic hyperglycemia (Table 3). Box and whisker plots corresponding to the entries of Table 3 are shown in Figure 1A–F. According to DeLong’s test, there were no significant AUC differences in SeptiCyte performance between patients with and without the stated condition.

We note several limitations to this analysis because of the small sample sizes. The AUC value of 1.0 for patients with acute cardiovascular disease should be considered imprecise because of low *N*. Additionally, while the uncorrected *p*-value for each individual comparison falls below the conventional cutoff for significance (*p* = 0.05), applying a Bonferroni correction causes some comparisons to fall above the adjusted cutoff for significance. Specifically, if we consider as a group the nine comorbidity/disease comparisons for which either the sepsis or SIRS subgroup contains <30 patients, then upon applying a Bonferroni correction, the *p*-values for diabetes and obesity now fall above the adjusted significance cutoff (*p*~0.05/9) and the *p*-value for chronic CVD becomes borderline significant.

Hyperglycemia—The cohort was stratified based on whether or not patients had hyperglycemia (≥200 mg/dL) (Figure 1A). A few patients with hypoglycemia were also noted, but their numbers were too small (14 sepsis, 3 SIRS) for reliable statistical analysis. Hyperglycemia included those patients with and without diabetes (as indicated in the physician notes). For patients without diabetes indicated, data were based on “Glucose.Min” or “Glucose.Max” values recorded over a 24 h period. The SeptiCyte RAPID performance for patients with diabetic hyperglycemia (AUC 0.73) appeared marginally lower than for all hyperglycemic patients (AUC 0.83) or for non-hyperglycemic patients (AUC 0.82). Note that although the *p*-value for sepsis vs. SIRS discrimination in the diabetic hyperglycemia subgroup (*p* ≈ 0.04) falls below the conventional cutoff (*p* = 0.05), after a Bonferroni correction, this *p*-value now falls above the adjusted significance cutoff (*p* = 0.05/9).

Impaired immunity—The cohort was stratified based on whether or not patients could be considered to have impaired immunity (Figure 1B). The category of impaired immunity included the use of immunosuppressants (including corticosteroids), adrenal insufficiency, splenectomy, asplenia, HIV/AIDS, and cancer. Of the 13 patients with cancer, no consideration was given to the type of cancer, whether the patients were being treated with cancer therapy, or the duration of cancer therapy. White blood cell counts (WBC, min) in patients with impaired immunity, as defined above, ranged from 300 to 37,000 cells/uL, and for patients with no such impaired immunity, the range was 300–52,000 cells/uL. SeptiCyte RAPID AUCs were 0.83 and 0.82 for patients with impaired immunity vs. immunocompetent, respectively.

Hypertension—The cohort was stratified based on whether hypertension was noted in the physician comments (Figure 1C). The AUC for differentiating sepsis from SIRS was 0.79 for hypertensive patients, and 0.83 for those without hypertension noted, a difference that was not significant according to DeLong’s test (*p* = 0.59).

Cardiovascular disease (CVD)—The cohort was stratified based on whether patients had been diagnosed with CVD (Figure 1D). CVD patients were further subdivided into those with acute (cardiac arrest) vs. chronic disease. The chronic CVD patients included those with congestive heart failure, aortic valve replacement, and bradycardia with cardiogenic shock. Of the patients with CVD, there were 16 with sepsis and 23 with SIRS. The remainder of the cohort (without CVD) consisted of 160 patients with sepsis and 220 with SIRS. We calculated AUC 0.85 for patients with CVD as opposed to AUC 0.82 for patients without CVD. This difference was deemed not significant by DeLong’s test (*p* = 0.63).

Kidney disease—The cohort was stratified into those with kidney disease (acute or chronic) vs. no kidney disease noted (Figure 1E). Kidney disease included patients with acute renal failure/injury vs. patients with chronic conditions (renal insufficiency/disease, end-stage renal disease, renal cell carcinoma, nephrolithiasis with bilateral ureteral stents, or chronic dialysis). AUCs for differentiating sepsis from SIRS were 0.82 (no kidney disease noted) and 0.83 for those with chronic kidney disease, a difference that was not significant.

Obesity—The cohort was stratified into obese (BMI ≥30 or obesity noted in physician comments) vs. BMI <30 or no obesity noted (Figure 1F). Obesity is a known risk factor for sepsis [32,33]. AUCs for differentiating sepsis from SIRS were 0.86 (obese patients) and 0.82 for non-obese patients; DeLong’s test indicates this AUC difference is not significant. Note that while the *p*-value for sepsis vs. SIRS discrimination in the obese subgroup (*p* ≈ 0.014) falls below the conventional cutoff (*p* = 0.05), after a Bonferroni correction, this *p*-value now falls above the adjusted significance cutoff (*p* = 0.05/9).

### 3.3. Source and Type of Infection

#### 3.3.1. Infection Source

**Figure 2:**
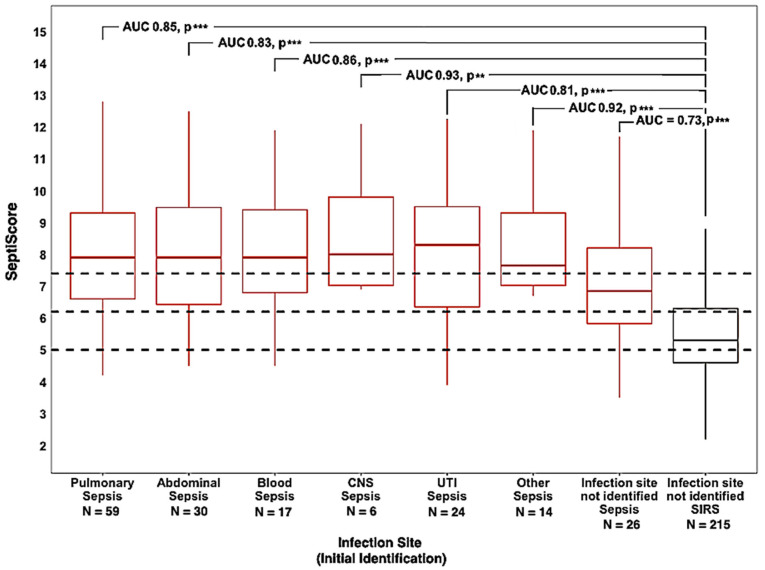
SeptiCyte RAPID performance stratified by infection source. The “other” group included the following (*N* per group): cellulitis (3), Fournier’s gangrene (1), hip arthroplasty (1), osteomyelitis (1), toe infection (1), sacral wound (1), post-surgical sternal wound (1), bladder/prostate abscess/peritonitis from cecum microperforation (1), influenza (1), tracheitis (1), erysipelas (1), skin or soft tissue necrotizing fasciitis (1). The dotted horizontal lines indicate the SeptiScore band boundaries as defined by Balk et al. [16]. Significance: *p* <= 0.01 **, *p* <= 0.001 ***.

Of the 176 sepsis patients in the cohort, 150 had an identified source of infection, specifically pulmonary (*N* = 59), abdominal (*N* = 30), blood (*N* = 17), central nervous system (CNS) (*N* = 6), urinary tract (UTI) (*N* = 24) and “other source” (*N* = 14, as detailed in the legend of Figure 2). There were 26 sepsis patients for whom an initial source of infection could not be identified (NI). In the cohort, 243 patients were retrospectively determined to have SIRS (deemed non-infectious); however, only 215 did not have a source of infection identified. Figure 2 presents box and whisker plots for subgroups of septic patients vs. the SIRS patients, with AUCs and *p*-values indicated. SIRS patients with an identified source of infection (4 abdominal, 5 CNS, 15 pulmonary, 2 urinary, 2 other) were excluded from the analysis. For all sepsis subgroups with identified sources of infection, the median SeptiScore was in Band 4 (highest sepsis probability), as defined in the banding scheme of Balk et al. [16]. For septic patients without an identified source of infection, the median SeptiScore was in Band 3. For the sepsis subgroups with sufficient numbers to allow for a reliable AUC comparison, DeLong’s test showed no significant differences (pulmonary vs. NI, *p* = 0.08; abdominal vs. NI, *p* = 0.21; UTI vs. NI, *p* = 0.35). This observation is addressed further in the Discussion.

#### 3.3.2. Infection Type

We previously determined that SeptiCyte RAPID performance was not affected by the Gram (+/−) category of the sepsis pathogen (see Section S9 of the supplement to Balk et al. [16]). In the present study, we extended this analysis to determine if different sepsis pathogens were associated with different sources of infection. The results of this analysis are provided in Supplementary Material, Section S2. To summarize, viral pathogens were more often detected in patients with pulmonary sepsis, which may reflect a bias in the types of pathogen identification tests ordered for this subgroup. *Staphylococcus aureus* was the most frequently isolated pathogen (19% of all pathogen detection events) and was mostly associated with pulmonary and blood sources of septic infection. Gram-negative pathogens, in particular *Escherichia coli* and *Pseudomonas aeruginosa*, were most isolated in urosepsis.

### 3.4. Therapeutic Interventions

We examined the performance of SeptiCyte RAPID after stratification along two treatment dimensions: either (+/−) pharmaceuticals (immunosuppressants, antibiotics, inotropes, vasopressors) or (+/−) mechanical ventilation. Pharmaceutical treatments were selected from a list of >400 drugs noted in the patient records. Further detail on the immunosuppressants, antibiotics, anti-neoplastics, inotropes, and vasopressors listed in patient records is provided in Supplementary Material, Section S3.

**Figure 3:**
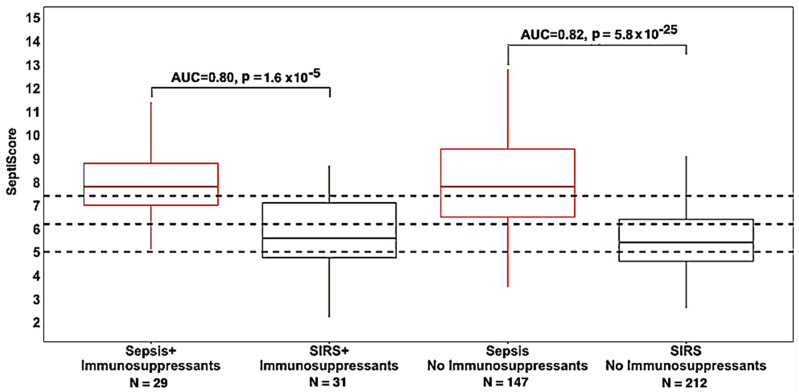
Box and whisker plot of SeptiCyte RAPID performance for patients treated vs. not treated with immunosuppressants. The dotted horizontal lines indicate the SeptiScore band boundaries as defined by Balk et al. [16].

The results in Table 4 and Figure 3 show that the use of a broad range of immunosuppressants did not affect the overall performance of SeptiCyte RAPID in our cohort. AUC values in patients treated with immunosuppressants (0.80) or not treated (0.82) were not statistically different (*p* = 0.75 by DeLong’s test).

**Figure 4:**
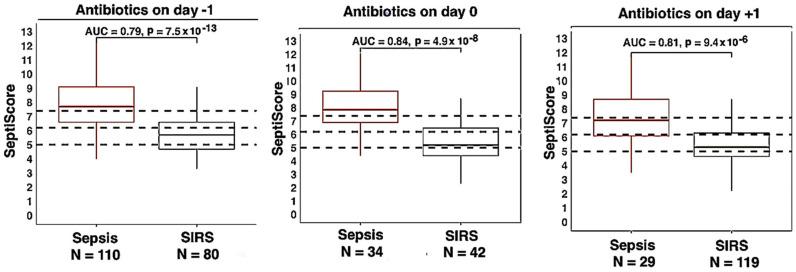
Box and whisker plots of the influence of antibiotic treatment (Abx) initiation time on SeptiCyte RAPID performance. The day of blood draw is defined as day 0. (**left**) treatment initiated −1 day to 0 days relative to blood draw; (**middle**) antibiotic treatment initiated on the same day as blood draw. (**right**) antibiotic treatment initiated +1 day after blood draw. The dotted horizontal lines indicate the SeptiScore band boundaries as defined by Balk et al. [16].

Patients treated with antibiotics (Abx) were subdivided into three groups based on when the antibiotic treatment was started relative to SeptiCyte RAPID blood sampling. In total, 190 patients (110 sepsis, 80 SIRS) were given antibiotics up to one day prior, 76 patients (34 sepsis, 42 SIRS) were given antibiotics on the same day, and 148 (29 sepsis, 119 SIRS) received antibiotics on the day following the blood sampling. SeptiCyte RAPID AUCs were not significantly affected by the timing of antibiotics over these time periods, with AUCs ranging from 0.79 to 0.84 (Figure 4). The purpose of this comparison was to determine if antibiotics interfered with SeptiCyte RAPID performance in a restricted time window from −1 day to +1 day relative to blood draw at day 0. We limited the analysis to this relatively narrow time window because a recent study [34] indicated that a reduction in SeptiScore is expected over the period of 2–6 days following initiation of antibiotics.

The use of vasopressors or inotropes had no evident effect on SeptiCyte RAPID performance (AUC 0.83 versus AUC 0.81 in the absence of vasopressors or inotropes, *p* = 0.60 by DeLong’s test). Similarly, SeptiScores in patients on mechanical ventilation (AUC 0.80) did not differ significantly from patients not on mechanical ventilation (AUC 0.84), *p* = 0.36 by DeLong’s test.

### 3.5. Phenotypic Subgrouping

The foregoing analyses have stratified patients on the basis of demographics, comorbidities and diseases, infection sources and types, and therapeutic interventions. Another complementary approach would be to examine clinical characteristics, such as vital signs, bloodwork parameters, and clinical chemistry test values, to determine if particular combinations of these phenotypic variables could reveal discrete strata within the patient cohort that would otherwise be masked or hidden. To address this possibility, we turned to the use of more sophisticated bioinformatics methods that have been developed to probe for within-group heterogeneity.

Our initial approach was based on principal component analysis (PCA). In this method, the dimensionality of the original dataset is reduced by constructing linear combinations of the most informative variables to define new vectors or ‘dimensions’ by which the two principal subgroups (sepsis, SIRS) can be maximally resolved. Variables that evidently have low information content are ignored or down-weighted in this step, which acts essentially as a filter. Then, a hierarchical clustering algorithm is applied, using the PCA vectors as input variables, to effect a separation into subgroups or clusters. This approach can, in some cases, reveal heterogeneity that might not be apparent otherwise.

#### 3.5.1. Phenotypic Subgrouping of the Septic Patients (*N* = 176) by PCA/HC

**Figure 5:**
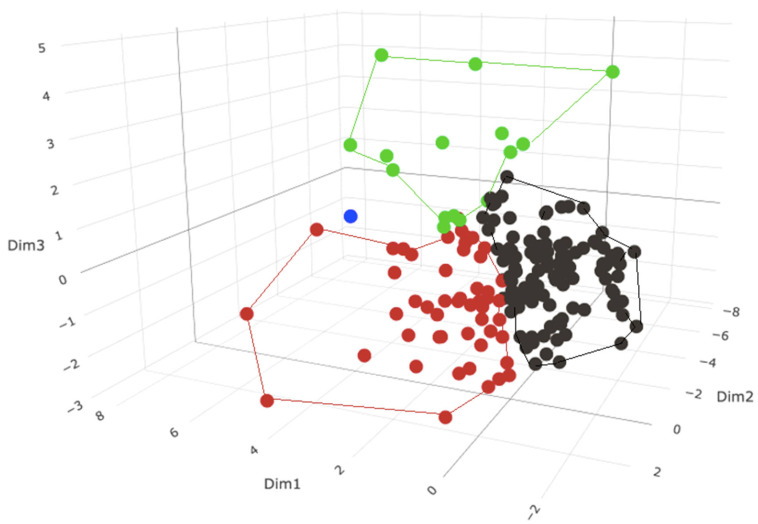
PCA plot of the sepsis group (*N* = 176) with superimposed HC using 16 phenotypic variables. In the plot, the peripheral points in each subgroup were used to define the cluster boundaries for that subgroup. Sepsis subgroup 1 (black) *N* = 110. Sepsis subgroup 2 (red) *N* = 50. Sepsis subgroup 3 (green) *N* = 15. Sepsis subgroup 4 (blue) *N* = 1.

We first conducted a PCA based on 16 pre-selected clinical variables and excluded SeptiScores and RPD determinations. The first two PCA dimensions captured 15.32% and 12.29%, respectively, of the total variation. A hierarchical clustering (HC) analysis was then performed on the PCA, which gave a separation into three major subgroups (Figure 5). There is additionally a single outlier patient, denoted as “subgroup 4” and represented by the blue point. This patient has a combination of elevated values for glucose (min and max), lactate, WBC (max), SOFA (liver component score), and lower values for temperature (min). Of note is that blood culture-positive patients did not cluster in any particular subgroup (not shown in the figure).

We next evaluated the performance of SeptiCyte RAPID for discriminating the SIRS group (*N* = 243) from each of the three sepsis subgroups defined by the PCA/HC analysis. The results are given in Table 5. DeLong’s test indicates that SeptiCyte RAPID performance is significantly better for subgroup 3 (AUC 0.93) than for subgroups 1 or 2 (AUC 0.81).

To identify the phenotypic variables that cause the three subgroups to differ—and in particular, to identify the variables that make subgroup 3 distinct from subgroups 1 and 2—we conducted an ANOVA. Phenotypic variables with greatest statistical significance (*p* < 0.01) are presented in Table 6. This analysis revealed that patients in subgroup 3 appeared the most seriously ill, as indicated by relatively higher SOFA, PCT, RR (min and max), HR (max), WBC (max), lactate values, and vasopressor use, and relatively lower MAP (min), glucose (min), platelets (min) and temperature (min). Of the 15 patients in subgroup 3, a total of 11 were clinically diagnosed with septic shock and 3 with severe sepsis based on Sepsis-2 definition, with no viral infections. Notably, we find no significant differences between the three sepsis subgroups with respect to the site of infection or type of infecting pathogen.

**Figure 6:**
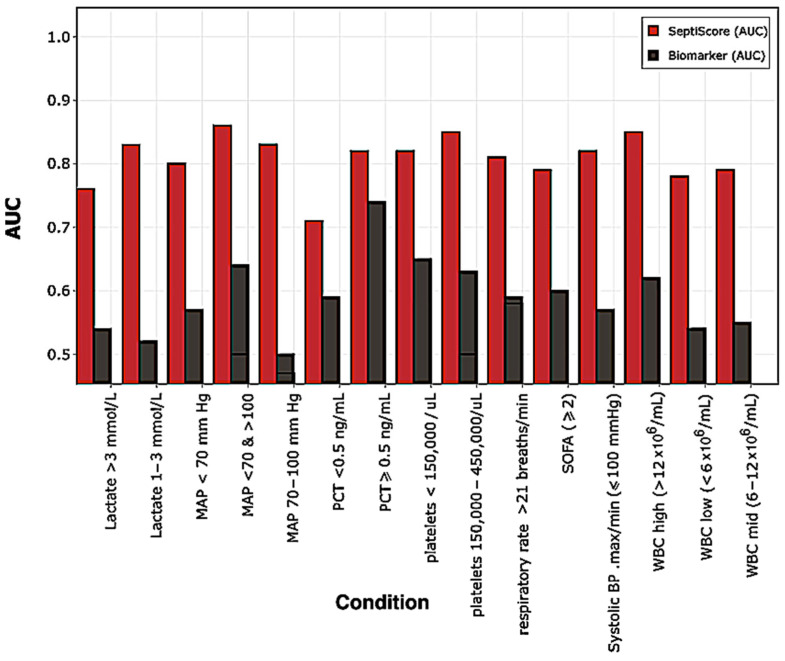
Performance for discriminating sepsis vs. SIRS in different phenotypic subgroups defined by individual driving variables in PCA/HC and other biomarkers used for sepsis adjudication. Red: SeptiScore. Black: driving variable. Additional details corresponding to this figure are presented in Supplement Table S5.

In the sepsis group, the PCA variables contributing greater than average (>6%) phenotypic variability for dimensions 1 and 2 were WBC (min and max), glucose (min and max), platelets (min), age, temperature (min), and MAP (min). We analyzed SeptiCyte RAPID performance in different subgroups defined by either these individual driving variables or other biomarkers used for sepsis adjudication (Figure 6). Similar SeptiCyte RAPID performance levels (AUC 0.79–0.85) were observed for most subgroups, indicating that SeptiCyte RAPID should still have utility regardless of the values of these driving variables. The highest SeptiScore AUCs (0.85) were found for patient subgroups having high WBC counts (>12 × 10^6^/mL) or normal platelet counts (150,000–450,000/uL). The lowest SeptiScore AUC (0.71) was found for the subgroup with PCT <0.5 ng/mL. This low PCT subgroup contained 32 sepsis patients who appeared less seriously ill than the high PCT (>0.5 ng/mL) subgroup, as indicated by a lower mean SOFA score and a lower proportion of mechanical ventilation. The low PCT sepsis subgroup also had fewer patients that were bacterial culture-positive (40.6%) and a higher percentage of patients that were viral-positive (31.3%), as compared to the high PCT subgroup, which was 66% bacterial culture-positive (*p* < 0.01) and only 15% viral-positive (*p* < 0.03). The detailed quantitative analysis corresponding to Figure 6 is presented in Supplementary Material, Section S5, Table S5. We note that the individual clinical parameters driving separation in the PCA were not themselves effective at discriminating sepsis vs. SIRS (AUC 0.5–0.6), consistent with the previous analysis of Balk et al. [16].

#### 3.5.2. Phenotypic Subgrouping of the Septic Patients (*N* = 176) by k-Means Clustering

Intrigued by the observation that the sepsis patient group could be cleanly resolved into three phenotypic subgroups by PCA/HC, we decided to also explore the use of an alternative stratification method, k-means clustering [30]. This is a widely used ‘classical’ method of clustering based on minimizing the internal (within-group) sums of squared deviations around group centroids (Euclidian distances). k-means clustering is recognized to be highly dependent on the initial assumption of *N*, the number of clusters. To determine *a priori* an optimal number of clusters, we relied on the silhouette method [31], which indicated that, for the sepsis dataset, the optimal number of clusters should be 2.

**Figure 7:**
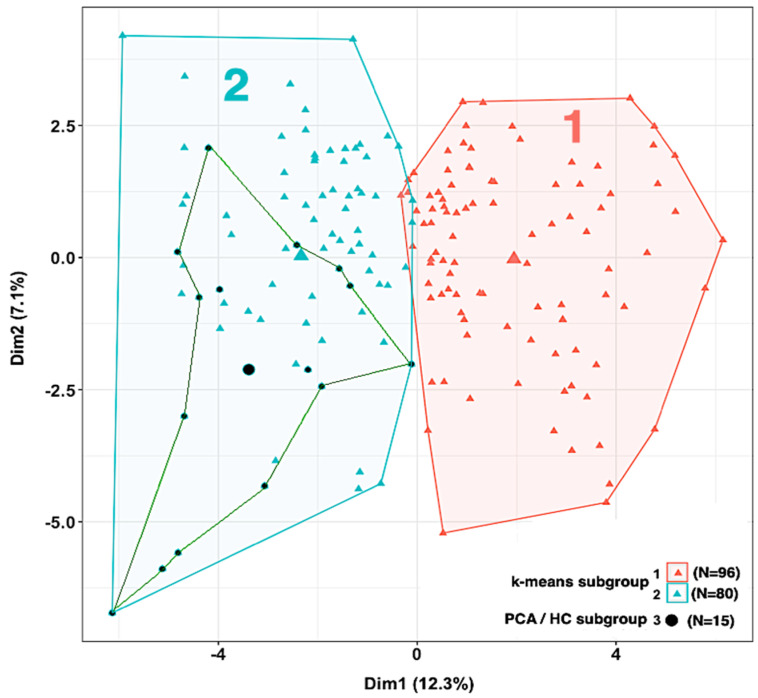
Sepsis subgroups 1 (red; *N* = 96) and 2 (blue; *N* = 80) identified by k-means clustering. The small, most seriously ill subgroup 3 from the PCA/HC analysis (black; *N* = 15) is contained entirely within k-means group 2. The large colored symbols indicate the centroids of the subgroups.

The results of this analysis are shown in Figure 7. The k-means clustering algorithm produced a very clear separation of the sepsis patients into the two subgroups. We next conducted an ANOVA to identify which of the phenotypic variables in the analysis provided the most significant discrimination between the two subgroups. The results of the ANOVA are presented in Table 7. (For vital signs, clinical chemistry measurements, and interventions, only those variables giving *p* < 0.01 are presented.) Interestingly, with the sole exception of viral infections being more prominent in subgroup 1, neither the site of infection nor pathogen type were significant drivers of the separation between the two k-means subgroups. Also, blood culture-positive patients did not cluster in either of the two subgroups (not shown in the figure). Compared to subgroup 1, subgroup 2 appeared more seriously ill, being distinguished by depressed temperature and MAP; elevated WBC, lactate, PCT, and SOFA; and increased administration of vasopressors and mechanical ventilation. Despite these clinical differences between subgroups, SeptiCyte RAPID AUCs for differentiating subgroup 1 sepsis patients (*N =* 96) and subgroup 2 sepsis patients (*N* = 80) from SIRS patients (*N =* 243) were 0.80 and 0.85, respectively.

Finally, we performed a Venn diagram analysis to determine overlap(s) between the subgroups identified by PCA/HC stratification vs. k-means stratification. We determined that all 15 of the patients classified as subgroup 3 in the PCA/HC analysis—patients resembling septic shock or severe sepsis according to the Sepsis-2 criteria—fell within k-means subgroup 2, as indicated in Figure 7.

## 4. Discussion

It is well known that patients with sepsis are heterogeneous with respect to clinical signs, response to therapy, and outcome. Factors contributing to this heterogeneity include patient demographics, comorbidities and diseases, infecting pathogen, locus of the infection, concurrent therapies, and disease progression and stage [35]. For maximum clinical utility, any sepsis diagnostic test should retain a high level of performance when confronted with these sources of variability. Accordingly, we investigated the performance of a commercial gene-expression-based sepsis test (SeptiCyte RAPID) across a broad range of patient characteristics. We demonstrated generally consistent and robust diagnostic performance of SeptiCyte RAPID in a heterogeneous, adult, critically ill patient population suspected of sepsis.

Demographics: We found no statistically significant differences in AUC for SeptiCyte performance in discriminating sepsis vs. SIRS with respect to age, sex, or ethnic/racial group when an appropriate number of cases per group were analyzed (Table 2). Upon closer examination, however, we did observe a race/ethnicity-based difference in SeptiScore performance for distinguishing *septic shock* from SIRS (AUC 0.83 for septic shock vs. SIRS in White patients; AUC 0.96 for septic shock vs. SIRS in Black patients; *p* < 0.003 by DeLong’s test; see Supplementary Material, Section S1). Higher SeptiScores for septic shock cases in Black as opposed to White patients could relate to genetics, environment, or a mixture of both. However, a number of studies suggest that genetic differences do not influence sepsis mortality or hospital length of stay once socioeconomic factors, such as the number of comorbidities and access to healthcare, are taken into account [36,37]. Without further data, including increased patient numbers and details on socioeconomic variables, it is not possible to definitively identify the factors contributing to the observed higher SeptiScores in Black vs. White patients with septic shock.

Comorbidities and diseases: Risk factors for sepsis include certain comorbidities, for example, diabetes [38,39,40], hypertension [40], chronic kidney disease [41,42], obesity [32,33], cancer [43], and cardiovascular disease [44]. Because SeptiCyte RAPID is based on the measurement of host immune response, comorbidities that involve the immune system could affect assay performance. AUC values for subgroups based on comorbidity and disease generally clustered around 0.82–0.83. One of the lowest SeptiCyte AUC values was observed for patients with diabetic hyperglycemia (AUC 0.73), but this was not significantly different (*p* = 0.51) from the AUC 0.82 observed for patients with no hyperglycemia noted (Table 3). Another low SeptiCyte AUC value was observed for patients with hypertension (AUC 0.79), but this was not significantly different (*p* = 0.59) from the AUC 0.83 observed for patients with no hypertension noted (Table 3). Despite the small sample sizes in some patient subgroups, these results suggest that certain patients suspected of sepsis need not be excluded from SeptiCyte RAPID testing due to the specific pre-existing comorbidities and diseases we were able to examine in our study cohort. Evaluation of SeptiCyte RAPID performance in patients stratified according to comorbidities and diseases would benefit from additional studies in an expanded patient cohort.

Type and site of infection: The immune status of leukocytes varies significantly depending upon the body compartment from which they are isolated [45] and the type of infection being responded to [46]. For example, in humans, an intravenous lipopolysaccharide (LPS) injection suppresses the ex vivo peripheral blood mononuclear cell (PMBC) response to LPS but primes that of alveolar macrophages [45]. Therefore, the primary site of infection in sepsis could be expected to influence the PBMC response and, hence, SeptiCyte RAPID results. To investigate this further, we compared SeptiCyte RAPID performance in patient subgroups stratified by the primary site of infection, including the categories lung, abdomen, central nervous system, urinary tract, blood (bacteremia), other identified site, and ‘site not identified’ (i.e., sepsis of unknown origin) (Figure 2). It may at first seem surprising that no statistically significant differences in SeptiCyte RAPID performance were observed across a broad range of identified infection sites. However, note that the sample source was peripheral blood rather than immune cells derived from the specific infection sites. We hypothesize that circulating blood leukocytes, by experiencing different microenvironments while traveling throughout the body, may reflect the “global immune status of patients” rather than specific immune mechanisms or responses occurring in a particular infected tissue [45]. AUC did appear lower (0.72) for patients with sepsis of unknown origin, which may relate to uncertainty in the retrospective diagnosis of patients in this subgroup [6,47].

Therapeutic interventions: immunosuppressants: —We did not observe any significant effect of immunosuppressant therapy on the performance of SeptiCyte RAPID (Table 4). However, this comparison covered a broad range of immunosuppressants, so no firm conclusions on the effect of any individual immunosuppressant on SeptiCyte RAPID can be drawn without performing a larger study with sufficient patient numbers. Regarding the glucocorticoid class specifically, it is known that short-term oral prednisone (4 days, 30 mg/day) produces a distinct blood gene expression signature in COPD patients and that neither PLAC8 nor PLA2G7 were differentially expressed when comparing pre-treatment patients to day 4 of treatment [48]. Further, PLAC8 and PLA2G7 are not known to be glucocorticoid-regulated genes [49]. This suggests that treatment with glucocorticoids does not affect peripheral blood gene expression of the two biomarkers of SeptiCyte RAPID and, therefore, is not expected to affect SeptiCyte RAPID results.

Interventional treatment: antibiotics: Although blood cultures are the ‘gold standard’ for confirming bacteremia, up to fifty percent of patients suspected of sepsis and admitted to the ICU have negative cultures [50], some of which could be caused by prior use of antibiotics [51]. Standard practice in patients suspected of sepsis is to take blood cultures prior to antibiotic administration to avoid the potential negative impact on the growth of organisms in blood culture. In this study, we show that the use of prophylactic antibiotics within 1 day prior to a patient presenting to the ICU does not affect SeptiCyte RAPID performance. Therefore, a clinician need not withhold antibiotics prior to taking a blood sample for SeptiCyte RAPID analysis. However, treatment with antibiotics outside of this timeframe would be expected to affect SeptiScores as patients recover in response to appropriate antibiotic treatment [34].

Phenotypic analysis: Of the bioinformatics methods available for in-depth analysis of complex datasets, we chose principal component analysis (PCA), hierarchical clustering (HC), and k-means clustering, as these are well developed theoretically and widely used. PCA was employed in the initial (upstream) phase of the analysis to reduce the dimensionality of our large dataset by identifying a limited number of variables containing most of the diagnostic information. The combination of PCA and HC [27,28,29] allows data to be projected into n-dimensional space using both numeric continuous variables and also categorical variables. Both types of variables would be used by clinicians in patient assessment, so this approach seemed to us to be well aligned with clinical practice.

We conducted phenotypic analyses along the lines described by Seymour et al. [3] on our sepsis patient cohort (*N* = 176). We identified three sepsis subgroups by PCA/HC and two sepsis subgroups by k-means clustering. Key driving variables separating the sepsis subgroups included WBC, platelets, MAP, glucose, platelets, age, and temperature. We also conducted the same type of analysis on the entire sepsis+SIRS dataset (*N* = 419). Similar to what we observed in the first analyses, these same phenotypic variables also resolved three subgroups in the PCA/HC analysis and two subgroups in the k-means analysis (Supplementary Material, Section S5). The separation between subgroups in either analysis appeared to be driven at least partly by the clinical severity of the sepsis response. Interestingly, neither the infection source nor the type of infecting pathogen was a significant factor, except that viral infections appeared to be less severe and more highly associated with sepsis of pulmonary origin.

Individual clinical variables that were identified as driving the PCA separation were assessed for their ability to discriminate sepsis from SIRS. Figure 6 compares the performance of these variables versus SeptiCyte RAPID. The results suggest that, in our study at least, patients with sepsis cannot easily be distinguished from those with SIRS using any of the individual clinical variables examined in this figure. In contrast, in subgroups defined by these driving variables, SeptiCyte RAPID differentiated sepsis from SIRS, with AUCs ranging from 0.71 to 0.86. This finding is consistent with a previous demonstration [16] showing that combinations of up to 14 clinical variables, including procalcitonin (PCT) and C-reactive protein (CRP), do not outperform SeptiCyte RAPID in discriminating sepsis from SIRS (see reference [16] for details). These results suggest that sepsis diagnostic tools that rely on these clinical variables alone (e.g., qSOFA or early warning scores) will be limited in their capacity to differentiate sepsis and SIRS patients. As such, it would seem the first step in a diagnostic workup would be to determine whether or not a patient had sepsis and then use the observed values of clinical parameters to guide downstream patient management.

The clinical variables we identified as “driving” the phenotypic separations in this study can be broadly mapped onto the phenotypes defined by Seymour et al. [3]: α, (systolic BP, limited use of vasopressors); β, (SOFA, chronic illness, renal dysfunction, age); γ (RR, WBC, PCT, platelets inflammation, and pulmonary dysfunction); and δ (systolic BP, lactate, shock). Further, Sinha et al. [8] have identified two subgroups in ARDS and septic shock termed “hypo-inflammatory” and “hyper-inflammatory”. Patients with hyper-inflammatory septic shock had higher rates of blood culture positivity, increased inflammatory markers, and poor outcomes (increased 28-day mortality and lower ICU-free days).

With respect to sepsis/SIRS discrimination, we observed similar SeptiCyte RAPID performance across our identified phenotypic classes and key driving variables. An important practical result is that SeptiCyte RAPID performance appears largely unaffected by phenotype or by key clinical variables used to define the phenotypes (Table 5, Table 6 and Table 7). The one exception to this statement is that higher SeptiCyte RAPID performance was observed in a sepsis patient subgroup (PCA/HC subgroup 3) containing an elevated proportion of patients exhibiting septic shock or severe sepsis according to the Sepsis-2 criteria. This subgroup is characterized by lower glucose, platelets, MAP, and temperature, as well as higher SOFA, RR, WBC count, and lactate. Such patients appeared to be readily identified by clinicians as requiring more aggressive treatment, as evidenced by 80% of this subgroup being administered vasopressors. Higher SeptiCyte RAPID performance in septic shock patients could provide clinicians with added confidence to differentiate such patients from those with non-infectious cardiogenic shock, thus enabling more appropriate therapies to be applied.

There are several limitations to this study. The first limitation is that the patients in our study cohort consisted of only 419 patients admitted to ICU and tested on the first day of ICU admission. The relatively limited size of our cohort may allow for the possibility of bias in recruitment or treatment regimes. Also, had the study included patients who were tested at times outside the first day of ICU admission, the relative performance estimates and factor sensitivities might have been different. A second limitation is that some of the substrata in our study cohort had low numbers, leading to wide confidence intervals on performance measures such as AUC. Additionally, the study cohort was not large enough to include quantitative analyses of the effects of other comorbidities or diseases, such as primary immunodeficiencies or HIV-1 infection, known to influence sepsis probability and/or severity. A third limitation is that all the patients were adults ≥18 years of age. We cannot say whether any differences in SeptiCyte RAPID performance occur in adolescents, children, infants, or neonates. A fourth limitation is that, with respect to race/ethnicity, the dataset was sufficient only to analyze the main classes of subjects (White, Black, Asian, Hispanic) from North American and European geographic regions and did not include other minorities or patients from other geographic regions. Future studies will need to be undertaken to establish performance in other racial/ethnic groups within North American and European populations, as well as racial/ethnic groups more generally from other areas of the world. A fifth limitation is that the metadata in our study was not sufficiently comprehensive or granular to allow a stratification according to socioeconomic factors, which are known to correlate with sepsis comorbidities [40].

Clinical identification of sepsis, even retrospectively, is characterized by significant diagnostic uncertainty [6,52]. This motivates the development of objective sepsis diagnostic tests [53,54]. In this study, we have shown that SeptiCyte RAPID provides consistent discrimination of sepsis vs. SIRS across many subgroups within a heterogeneous adult ICU patient population suspected of sepsis.

SeptiCyte RAPID has regulatory clearance in the USA, Europe, and Australia to aid in the diagnosis of sepsis in adults. Clinician feedback has suggested clinical utility, impacting both antibiotic stewardship and diagnostic stewardship, in the following ways. (1) Because SeptiCyte RAPID has a fast (one hour) turnaround time, results are obtained concurrently with white cell counts and clinical chemistry data, which can facilitate early correct decision making. (2) A high SeptiScore (Band 4), in combination with other clinical and laboratory results, provides evidence to confirm an initial clinical suspicion of sepsis. This in turn helps to justify an early appropriate administration of a sepsis bundle, including antibiotics, resulting in lower morbidity and mortality [55,56]. (However, as the test does not differentiate pathogen type, but only the presence or absence of sepsis, tailoring antibiotic regimens is not possible.) (3) A low SeptiScore (Band 1), in combination with other clinical and laboratory results, provides evidence for a low sepsis probability, justifying the withholding of a sepsis bundle (including withholding antibiotics), as per the Surviving Sepsis Management Guidelines [57]. This also would allow clinicians to consider alternate non-infectious etiologies in their diagnostic workup. (4) Clinicians have also expressed interest in extending using SeptiCyte RAPID in select patient groups, including pediatric patients, post-operative patients, and patients with burns, trauma, or neutropenic fever. Further studies involving larger numbers of patients will be required to confirm the robustness of SeptiCyte RAPID in broader or more specific populations.

## 5. Conclusions

We conducted a stratification analysis of SeptiCyte RAPID data from the pivotal studies MARS, VENUS, and NEPTUNE (*N* = 419). SeptiCyte RAPID demonstrated consistent performance for discrimination of sepsis vs. SIRS, using AUC as the performance measure, across a heterogeneous adult, critically ill patient population when stratified by key demographic, clinical, microbiological, and interventional parameters. This helps to support a general clinical utility claim for SeptiCyte RAPID. Stratification analysis with phenotypic variables was also conducted, leading to the identification of distinct patient subgroups on the basis of combinations of phenotypic and clinical variables. One subgroup appeared to be mainly populated by patients exhibiting more severe sepsis phenotypes, including septic shock phenotypes. SeptiCyte RAPID exhibited consistently strong performance (AUC 0.81–0.93) across phenotypic subgroups. Phenotypic stratification may be useful for resolving sepsis patients into subgroups based on clinical severity, which in turn could suggest appropriate therapeutic interventions. Work is currently in progress to examine additional patient cohorts and datasets using the stratification methods described herein.

## Figures and Tables

**Table 1:** Agreement between global mean imputation and group mean imputation methods, as applied to missing data in PCA + HC cluster analysis.

		Global Mean Imputation		
		Cluster 1	Cluster 2	Cluster 3
Group Mean Imputation	Cluster 1	251	0	1
	Cluster 2	0	29	0
	Cluster 3	21	0	117

The % cluster agreement = (251 + 29 + 117)/419 = 397/419 = 94.75% and Cohen’s kappa = 0.898.

**Table 2:** SeptiCyte RAPID performance for sepsis/SIRS discrimination for patients stratified by sex, age, or race/ethnicity. Performance quantified by AUC and *p*-value using forced adjudication. AUC confidence intervals (CI) by the formula of Hanley and McNeil [20] and comparison of AUC values by DeLong’s test [21]. All *p*-values remained significant (*p* < 0.05/8) after applying a Bonferroni correction to all subgroups.

Condition	*N* Sepsis	*N* SIRS	AUC	AUC 95% CI	*p*-Value	DeLong’s *p*-Value
Male (M)	95	137	0.81	0.75–0.87	1.4 × 10^−15^	M vs. F: 0.52
Female (F)	81	106	0.84	0.78–0.90	1.6 × 10^−15^	
Age <60 years	77	138	0.83	0.77–0.89	3.9 × 10^−14^	Age (<60) vs. (≥60): 0.82
Age ≥60 years	99	105	0.82	0.76–0.88	5.7 × 10^−16^	
Black (B) *	45	70	0.85	0.77–0.93	5.9 × 10^−11^	B vs. W: 0.30 B vs. H: 0.21 B vs. A: 0.62 W vs. H: 0.03 W vs. A: 0.23 H vs. A: 0.63
White (W) *	108	146	0.80	0.74–0.86	7.2 × 10^−16^	
Hispanic (H) *	10	12	0.93	0.81–1.05	0.00014	
Asian (A) *	10	11	0.89	0.74–1.04	0.0013	

* Seven patients were unclassified as to race/ethnicity, so they were left out of this analysis.

**Table 3:** SeptiCyte RAPID performance stratified by comorbidities and disease. Abbreviations: HG, hyperglycemia; II, impaired immunity; HT, hypertension; CVD, cardiovascular disease; KD, kidney disease. AUC confidence intervals (CI) by the formula of Hanley and McNeil [20]. For small sample comparisons, the *p*-value was calculated using both the *t*-test (T) and the Mann–Whitney U test (U).

Stratification	*N* Sepsis	*N* SIRS	AUC	AUC 95% CI	*p*-Value	Delong’s *p*-Value
Hyperglycemia (HG)	50	78	0.83	0.76–0.92	5.3 × 10^−10^	HG vs. no HG noted: 0.76 HG_d_ vs. no HG noted: 0.51 HG_d_ vs. HG_¬d_: 0.37
Diabetic hyperglycemia (HG_d_)	10	16	0.75	0.51–0.93	0.035 (T) * 0.038 (U) *	
Diabetic hyperglycemia not noted (HG_¬d_)	40	62	0.85	0.77–0.93	3.7 × 10^−9^	
No HG noted	126	165	0.82	0.76–0.86	5.8 × 10^−21^	
Impaired immunity (II)	27	32	0.83	0.72–0.94	6.8 × 10^−6^	II vs. no II noted: 0.84
No impaired immunity noted	149	211	0.82	0.77–0.87	4.9 × 10^−25^	
Hypertension (HT)	24	26	0.79	0.66–0.92	1.8 × 10^−4^	HT vs. no HT noted: 0.59
No hypertension noted	152	217	0.83	0.78–0.88	1.0 × 10^−26^	
Cardiovascular disease (CVD)	16	23	0.85	0.72–0.98	1.7 × 10^−4^	CVD vs. no CVD noted: 0.63
Cardiovascular disease (acute)	7	6	1.0	0.75–1.0	0.0031 (T) 0.0034 (U)	
Cardiovascular disease (chronic)	9	17	0.81	0.62–1.00	0.0046 (T) 0.0105 (U) *	
No cardiovascular disease noted	160	220	0.82	0.78–0.86	1.9 × 10^−26^	
Kidney disease (KD)	33	34	0.83	0.73–0.93	5.6 × 10^−7^	
Kidney disease (acute)	13	13	0.86	0.70–1.00	0.0012 (T) 0.0023 (U)	KD vs. no KD noted: 0.89
Kidney disease (chronic)	20	21	0.83	0.70–0.96	0.0001 T) 0.0004 (U)	
No kidney disease noted	143	209	0.82	0.77–0.87	7.4 × 10^−24^	
Obesity (BMI ≥30)	6	14	0.86	0.66–1.06	0.0141 (T) * 0.0135 (U) *	Obesity vs. no obesity noted: 0.63
No obesity noted	170	229	0.82	0.78–0.86	1.8 × 10^−28^	

* No longer statistically significant under an adjusted cutoff (*p* = 0.05/9) from a Bonferroni correction applied to the nine strata with <30 patients per sepsis or SIRS subgroup.

**Table 4:** SeptiCyte RAPID performance stratified by therapeutic interventions. Abbreviations: Abx, antibiotics; I, inotropes; IS, immunosuppressants; MV, mechanical ventilation; V, vasopressors. AUC confidence intervals (CI) by the formula of Hanley and McNeil [20] and comparison of AUC values by DeLong’s test [21].

Therapeutic Intervention	*N* Sepsis	*N* SIRS	AUC (95% CI)	Sepsis vs. SIRS *p*-Value	DeLong’s *p*-Value
Immunosuppressants (IS)	29	31	0.80 (0.67–0.91)	1.6 × 10^−5^	IS vs. no IS: 0.75
No immunosuppressants	147	212	0.82 (0.77–0.87)	5.8 × 10^−25^	
Antibiotics (Abx) 1 day prior to SeptiScore	110	80	0.79 (0.73–0.85)	7.5 × 10^−13^	Abx prior vs. on = 0.46 Abx on vs. after = 0.71 Abx after vs. prior = 0.79
Antibiotics same day as SeptiScore	34	42	0.84 (0.75–0.93)	4.9 × 10^−8^	
Antibiotics 1 day after SeptiScore	29	119	0.81 (0.71–0.91)	9.4 × 10^−6^	
Vasopressors (V) or inotropes (I)	81	76	0.83 (0.77–0.89)	2.3 × 10^−14^	V/I vs. no V/I: 0.60
No vasopressors (V) or inotropes (I)	95	167	0.81 (0.75–0.87)	4.8 × 10^−15^	
Mechanical ventilation (MV)	62	91	0.80 (0.72–0.88)	3.1 × 10^−10^	MV vs. no MV: 0.36
No mechanical ventilation	114	152	0.84 (0.79–0.89)	5.4 × 10^−21^	

**Table 5:** SeptiCyte RAPID performance in the three subgroups defined by PCA + HCC analysis.

Comparison	*N* Sepsis	*N* SIRS	AUC	Sepsis vs. SIRS *p*-Value	DeLong’s *p*-Value
Sepsis PCA subgroup 1 vs. SIRS	110	243	0.81	6.9 × 10^−19^	1 vs. 2 *p* = 0.95 1 vs. 3 *p* = 0.015 2 vs. 3 *p* = 0.035
Sepsis PCA subgroup 2 vs. SIRS	50	243	0.81	4.5 × 10^−9^	
Sepsis PCA subgroup 3 vs. SIRS	15	243	0.93	2.2 × 10^−6^	

Subgroup 4 only had 1 patient, so it was not included in this table.

**Table 6:** Phenotypic characteristics of patients in the three subgroups from the PCA/HC analysis). *p*-values were calculated by one-way ANOVA for quantitative variables and by a proportions test for categorical variables.

Characteristic	Missing Values (%)	Subgroup 1 (*N* = 110), Median (IQR)	Subgroup 2 (*N* = 50), Median (IQR)	Subgroup 3 (*N* = 15), Median (IQR)	*p*-Value
Vital Signs					
Temperature (Min)	24 (14%)	36.3 (35.5–36.7)	36.0 (35.3–36.7)	35.4 (33.5–35.7)	<0.001
Heart rate (Max)	0 (0%)	113 (102–128)	128 (114–138)	138 (122–156)	<0.001
Mean arterial pressure (Min)	2 (1%)	62 (55–74)	59 (48–65)	50 (45–57)	0.006
Respiratory rate (Max)	53 (30%)	27 (24–31)	27 (23–31)	37 (33–43)	<0.001
Respiratory rate (Min)	56 (32%)	20 (14–24)	17 (12–23)	32 (15–36)	<0.001
Clinical Parameters					
Glucose (Min)	5 (3%)	123 (98–160)	129 (108–160)	90 (65–115)	0.009
WBC (Max)	7 (4%)	12 (8–15)	21 (18–29)	23 (18–29)	<0.001
WBC (Min)	5 (3%)	9 (6–12)	18 (15–23)	14 (8–23)	<0.001
PCT	27 (15%)	3 (0–17)	5 (2–13)	36 (14–130)	<0.001
Lactate	47 (27%)	2.0 (1.45–3.15)	2.4 (1.60–3.40)	9.3 (4.13–11.88)	<0.001
Platelets (Min)	52 (30%)	145 (102–212)	309 (238–367)	80 (53–221)	<0.001
SOFA	19 (11%)	6 (4–9)	6 (4–8)	11 (7–14)	<0.001
Vasopressor (Y/N)	-	42 Y (38%)	20 Y (40%)	12 Y (80%)	0.008
Infection Site *					1 vs. 2, 1 vs. 3, 2 vs. 3 (proportions test)
Pulmonary		34 (30.9%)	20 (40%)	4 (26.7%)	0.26, 0.73, 0.35
Abdominal		15 (13.6%)	10 (20%)	5 (33.3%)	0.30, 0.05, 0.29
Blood		12 (10.9%)	4 (8.0%)	1 (6.7%)	0.57, 0.23, 0.33
CNS		6 (5.5%)	0 (0%)	0 (0%)	0.08, 0.33, ND
Urinary tract		13 (11.8%)	8 (16%)	3 (20%)	0.47, 0.37, 0.13
Other		9 (8.2%)	4 (8.0%)	1 (6.7%)	0.97, 0.84, 0.87
Site not identified **		21 (19.1%)	4 (8.0%)	1 (6.7%)	0.07, 0.24, 0.87
Pathogens **					1 vs. 2, 1 vs. 3, 2 vs. 3 (proportions test)
Viral (including coinfections)		23 (21%)	6 (12%)	0 (0%)	0.17, 0.05, 0.16
E*scherichia coli*		11 (10%)	5 (10%)	2 (13.3%)	1.00, 0.70, 0.72
*Staphylococcus aureus*		18 (16.4%)	15 (30%)	2 (13.3%)	0.05, 0.76, 0.20
*Streptococcus*		6 (5.5%)	3 (6%)	2 (13.3%)	0.90, 0.25, 0.36
*Enterococcus*		4 (3.6%)	3 (6%)	0 (0%)	0.49, 0.46, 0.34

* Site of infection was not apparent or identified at time of enrollment or within 24 h of ICU admission, according to study investigator(s). ** Percentages are calculated column-wise.

**Table 7:** Phenotypic variables contributing to separation between the two sepsis subgroups in the k-means analysis of Figure 6. *p*-values were calculated with 1-way ANOVA for quantitative variables and with Pearson’s chi-squared test or two-sample proportions test for categorical variables. For vital signs, clinical chemistry measurements, and interventions, only those variables giving *p* < 0.01 are presented.

Characteristic	Missing Values (%)	Subgroup 1 (*N* = 96), Median (IQR)	Subgroup 2 (*N* = 80), Median (IQR)	*p*-Value *
Vital Signs				
Temperature (Min)	24 (14%)	36.4 (36.0–36.8)	35.3 (34.4–35.8)	<0.001
Mean arterial pressure (Min)	2 (1%)	67 (57–78)	55 (46–61)	<0.001
Respiratory rate (Max)	53 (30%)	25 (23–31)	29 (25–35)	0.01
Clinical Chemistry				
WBC (Max)	7 (4%)	12 (8–18)	18 (14–25)	<0.001
WBC (Min)	5 (3%)	10 (6–14)	14 (9–19)	<0.001
Lactate	47 (27%)	2.10 (1.45–3.00)	3.00 (1.83–5.85)	<0.001
GCS < 15 (qSOFA component)	25 (14%)	18 (18.8%)	42 (52.5%)	<0.001
qSOFA ≥ 2	-	22 (23%)	68 (85%)	<0.001
qSOFA < 2	-	62 (65%)	8 (10%)	<0.001
SOFA	19 (11%)	4 (2–6)	9 (7–12)	<0.001
PCT_binary (>0.5 ng/mL)	27 (15%)	52 (64%)	65 (96%)	<0.001
Interventions				
Vasopressors used	-	15 (16%)	60 (75%)	<0.001
Mechanical ventilation	-	18 (19%)	44 (55%)	<0.001
Infection Site *				
Pulmonary	-	36 (37.5%)	23 (28.8%)	0.22
Abdominal	-	13 (13.5%)	17 (21.2%)	0.18
Blood	-	13 (13.5%)	4 (5%)	0.06
Central nervous system (CNS)	-	4 (4.2%)	2 (2.5%)	0.54
Other	-	5 (5.2%)	9 (11.2%)	0.14
Urinary tract infection (UTI)	-	9 (9.4%)	15 (18.8%)	0.07
Site not confirmed initially	-	16 (16.7%)	10 (12.5%)	0.44
Pathogens *				
Viral (including coinfections)	-	23 (24%)	6 (7.5%)	0.006
*Escherichia coli*	-	8 (8.3%)	10 (12.5%)	0.36
*Staphylococcus aureus*	-	20 (20.8%)	15 (18.8%)	0.74
*Streptococcus*	-	5 (5.2%)	6 (7.5%)	0.53
*Enterococcus*	-	4 (4.2%)	3 (3.8%)	0.89

* Percentages are calculated column-wise.

## Data Availability

The datasets used and/or analyzed during the current study are available from the corresponding authors upon reasonable request.

## Supplementary Materials

The following supporting information can be downloaded at https://www.mdpi.com/article/10.3390/jcm13206044/s1. References [20,21,22,23,24,58,59,60,61,62,63,64,65,66] are cited in Supplementary Material.
